# Aortic calcification correlates with pseudoaneurysm or penetrating aortic ulcer of different etiologies

**DOI:** 10.1038/s41598-023-49429-y

**Published:** 2024-01-02

**Authors:** Siting Li, Haoxuan Kan, Zhili Liu, Rong Zeng, Jiang Shao, Yuexin Chen, Wei Ye, Yuehong Zheng

**Affiliations:** Department of Vascular Surgery, Department of State Key Laboratory of Complex Severe and Rare Diseases, Peking Union Medical College Hospital, Chinese Academy of Medical Sciences and Peking Union Medical College, Beijing, China

**Keywords:** Aortic diseases, Calcification

## Abstract

Chronic risk factors for pseudoaneurysm (PSA) or penetrating aortic ulcer (PAU) have not been fully clarified. This study aims to evaluate the association of aortic calcification with PSA or PAU of different etiologies. Totally 77 pseudoaneurysms, 80 PAU, and 160 healthy controls (HCs) were retrospectively included, of which 30 were infected, 34 were immunological, and 93 were atherosclerotic etiologies. The aortic calcification status, position of aortic tears/ulcers, and risk factors for disease or acute aortic syndrome (AAS) were identified. Atherosclerotic patients aged more than 65 and infective patients aged more than 60 had significantly higher calcification scores. The immunological group had a lower level of calcification in the infrarenal aorta. For patients of infective or atherosclerotic etiology, 60% (18/30) and 60.22% (56/93) of the tears/ulcers occurred at the aortic parts with the highest level of calcification. Patients with longitudinal calcification exceeding 1/3 of the aortic arch had an increased risk of acquiring diseases (OR = 13.231). The presence of longitudinal calcification of the descending aorta or cross-sectional calcification of the infrarenal aorta increased the risks of acquiring diseases (OR = 8.484 and 8.804). After adjusting for age, longitudinal calcification of the descending aorta exceeding 1/3 length was found to be associated with AAS (OR = 4.662). Tears/ulcers of pseudoaneurysm and PAU were both generally found at the part of the aorta with most calcification. Distinct aorta calcification characteristics were observed for lesions of different etiologies. Longitudinal thoracic and cross-sectional infrarenal abdominal aortic calcification increased the risk of acquiring diseases, and descending aortic calcification was associated with symptomatic patients.

## Introduction

Aortic pseudoaneurysm is defined as a dilatation of the aorta leading to disruption from the aortic wall layers and being only contained by the periaortic connective tissue^1^. Penetrating aortic ulcer (PAU) refers to a pathological characteristic where ulceration in the aortic wall deepens through the elastic lamina into the media layer^2^. Both pseudoaneurysm and PAU can develop an acute process with clinical manifestations, which also fall into the category of acute aortic syndrome (AAS). Traumatic, iatrogenic, infected, inflammatory, and atherosclerotic etiologies are all potential causes of pseudoaneurysm or PAU^3^. However, chronic risk factors for the diseases have not been fully clarified.

Calcium is often deposited in the coronary arteries as well as the aorta and its branches. It was hypothesized to be a hallmark of atherosclerotic plaque evolution^4^. Vessel calcification was strongly age-related and was observed to be associated with the occurrence, progression, and prognosis of cardiovascular events^5–9^. Aortic calcification was also present in pseudoaneurysm and PAU^10,11^. However, to the best of our knowledge, no studies have specifically reported the use of computed tomography (CT) to evaluate the distribution and severity of arterial calcification in patients with PAU or pseudoaneurysm.

In this study, we evaluate the association of aortic calcification with pseudoaneurysm or PAU of different etiologies using a scoring system adapted from previous studies^5,12,13^. The relationship between the calcification of different aortic parts and the position of aortic tears/ulcers was explored, and risk factors for disease or AAS were identified.

## Methods

### Patient inclusion and information collection

From 2014 to 2022, patients diagnosed with aortic pseudoaneurysm and penetrating aortic ulcer at Peking Union Medical College Hospital (PUMCH) were consecutively included. Patients with traumatic or iatrogenic history were excluded. Controls who had received chest-abdomen-pelvis CT for health examination were also included and matched with age and sex. Controls with a history of known inflammatory conditions or a recent history of infection were excluded. Infected etiology was defined for patients with concomitant aortic lesions, symptoms of infection, and positive blood culture or serological test of pathogens. Immunological etiology included patients priorly diagnosed with systematic vasculitis or systematic lupus erythematosus. Acute symptomatic patients included chest or abdominal pain, fever, dysphagia, and hoarseness within 14 days. Indications for intervention included symptomatic (recurrent and refractory pain, hemodynamic instability) and complicated (rapidly growing aortic ulcer, associated periaortic hematoma, signs of aortic rupture) PAU^14,15^. An endovascular-first approach was generally selected regardless of etiologies.

Medical history, intervention process, and clinical images were extracted from a prospectively maintained electronic medical records system. Most of the patients received at least one enhanced CT (1 mm), or CT angiography (0.5 mm, 0.75 mm, or 1 mm) before diagnosis, and a few received unenhanced CT (5 mm). The place of aortic tear or ulcer was identified for patients with pseudoaneurysm or PAU. The study was approved by the Peking Union Medical College Hospital Institutional Review Board (Approval number S-K1640, date 2021-5-19) and fulfilled the ethical guidelines of the declaration of Helsinki. Patients’ written informed consent was acquired.

### Calcification evaluation

**Figure 1:**
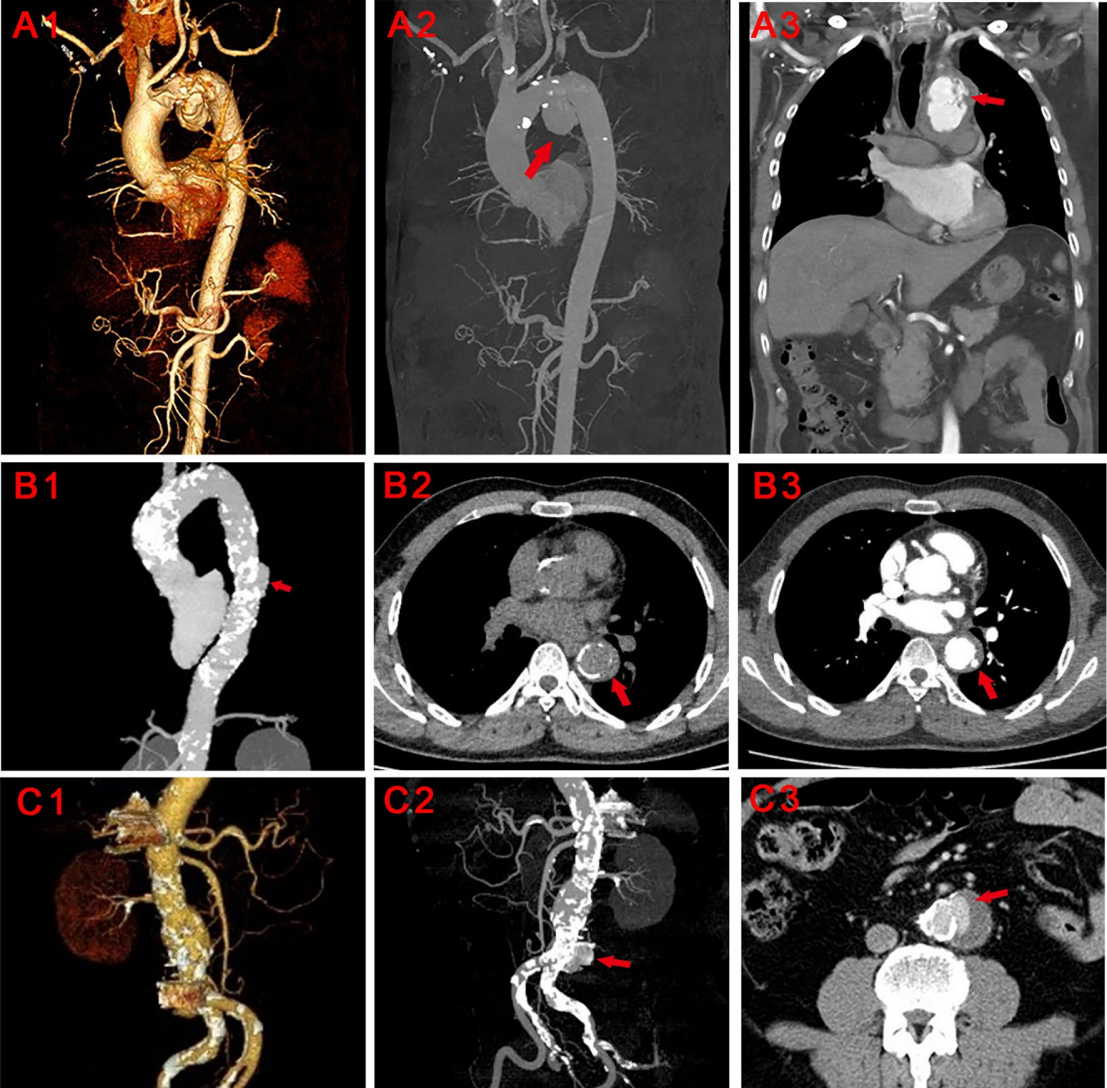
Examples of PAU/pseudoaneurysm evaluation. A1-A3: Example of PAU at aortic arch. Red arrows indicate the place of ulcer. B1-B3: Example of PAU at desceding aorta. Red arrows indicate the place of ulcer. C1-C3: Example of pseudoaneurysm at infrarenal abdominal aorta. Red arrows indicate the place of aortic tear.

The aorta was divided into five parts: the ascending aorta (valve to right subclavian), aortic arch (to the plane of Ludwig), descending thoracic aorta (to diaphragm), visceral aorta, and infrarenal aorta (10 mm from the lowest renal artery to iliac bifurcation). The calcification status of each part was evaluated with a scoring system adapted from previous reports^5,12,13^. Briefly, each aortic part was divided longitudinally into 3 even parts, and the percentage of scan planes showing calcification among the total planes of each aortic part was calculated. For each cross-sectional plane, the aorta was divided into 4 parts and calculated with the percentage of calcification. A calcification score of 0–3 and 0–4 was given respectively for longitudinal and cross-sectional calcification. The total calcification score was the summary of longitudinal and cross-sectional results (i.e. totally 0–15 and 0–20 points for longitudinal and cross-sectional aorta). Examples were illustrated in Fig. 1. The threshold for calcification was defined by Agatston^12^. Two trained observers evaluated all images independently and an additional observer was consulted for disagreement (approximately 8% of all cases).

### Statistical analysis

Data were analyzed using SPSS software (version 26.0) and R (version 4.0.2). Categorical variables were compared using the chi-square or Fisher exact test. Continuous variables were compared using the unpaired Student *t* test, one-way ANOVA test, or Mann–Whitney *U* test, if appropriate. A p value < 0.05 was considered statistically significant for all analyses. For logistic regression, univariate analysis was first performed to identify possible predictors. Variables achieving p < 0.10 were entered into a multivariable model using the backward conditional method. Odds ratio (OR) with 95% confidence interval (CI) were shown.

## Results

### Patient characteristics

A total of 77 pseudoaneurysm, 80 PAU, and 160 healthy controls were included in the study (Supplemental Table 1). All infected and most immunological lesions (22/34) were pseudoaneurysms, while PAU consisted of atherosclerotic (85%) or immunological (15%) etiologies. Pseudoaneurysm and PAU patients were grouped according to the etiology of aortic lesions (Table 1). There were 30 infected, 34 immunological, and 93 atherosclerotic patients in each group. The average age was 61.30 ± 9.89 for the infected, 42.26 ± 17.89 for the immunological, 66.81 ± 9.93 for the atherosclerotic, and 67.64 ± 0.63 for the healthy groups. Symptoms were more present in the infected (96.67%) and immunological groups (64.41%) (p < 0.001), and disease courses were also shorter for the two groups (p = 0.007). The immunological group had a lower percentage of diabetes (p = 0.005), dyslipidemia (p = 0.001), and smoking (p = 0.003) compared to other disease groups. Regarding intervention, endovascular repair (76.67%) was the most common choice for the infective group, conservative observation (61.76%) was the most common choice for the immunological group, and both endovascular (49.46%) and observation (46.24%) were common choices for the atherosclerotic patients.

**Table 1:** Characteristics of PAU or pseudoaneurysm patients with different etiologies.

	Infected (n = 30)	Immunological (n = 34)	Atherosclerotic (n = 93)	Health (n = 160)	p
Age, (mean years ± SD)	61.30 ± 9.89	42.26 ± 17.89	66.81 ± 9.93	67.64 ± 0.63	< 0.001
Sex, male n (%)	24 (80.00)	27 (79.41)	76 (81.72)	129 (80.63)	> 0.99
Symptom*, n (%)	29 (96.67)	22 (64.71)	33 (35.48)	NA	< 0.001
Disease course, (mean months ± SD)	1.77 ± 0.30	4.74 ± 1.83	9.28 ± 2.24	NA	0.007
Hypertension, n (%)	17 (56.67)	14 (41.18)	72 (76.34)	77 (48.13)	< 0.001
Prior coronary heart disease, n (%)	2 (6.67)	7 (20.59)	43 (46.24)	28 (17.50)	< 0.001
Prior stroke, n (%)	3 (10.00)	5 (14.71)	21 (22.58)	21 (13.13)	0.181
Diabetes Mellitus, n (%)	10 (33.33)	3 (8.82)	19 (20.43)	56 (35.00)	0.005
Dyslipidemia, n (%)	5 (16.67)	4 (11.76)	31 (33.33)	66 (41.25)	0.001
Ever smoke, n (%)	19 (63.33)	15 (44.12)	56 (60.22)	63 (39.38)	0.003
Ever drink, n (%)	12 (40.00)	6 (17.65)	32 (34.41)	59 (36.88)	0.164
Peripheral artery disease, n (%)	9 (30.00)	30 (88.24)	72 (77.42)	85 (53.13)	< 0.001
Operation					0.003
Open, n (%)	2 (6.67)	2 (5.88)	4 (4.30)	NA	
Endovascular, n (%)	23 (76.67)	11 (32.35)	46 (49.46)	NA	
Observation, n (%)	5 (16.67)	21 (61.76)	43 (46.24)	NA	

*SD* standard deviation.

*Symptoms included pain, fever, dysphagia, and hoarseness.

### Calcification and ulcer characteristics

**Figure 2:**
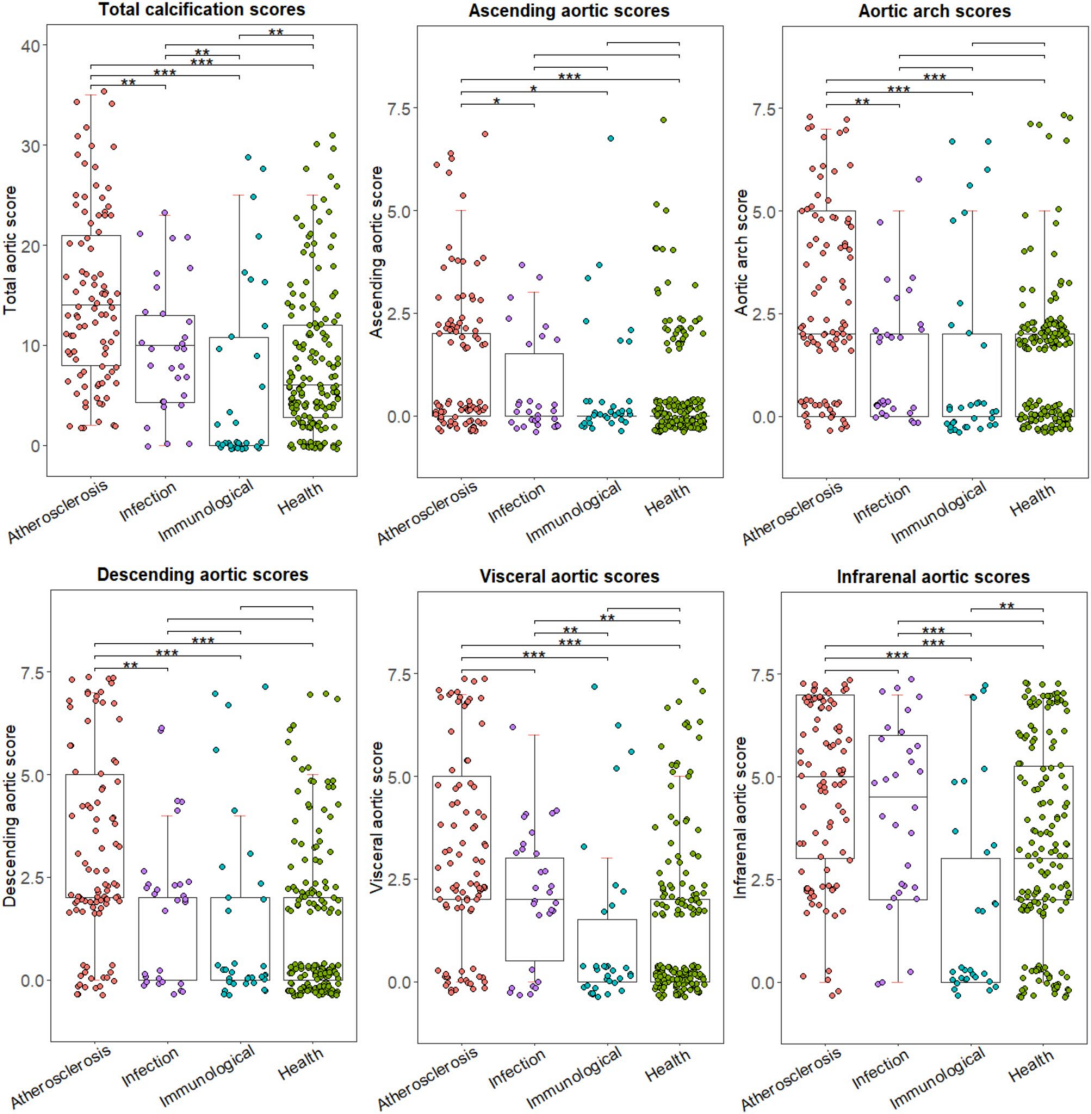
Calcification score of different aortic parts. The calcification score of total and each aortic part is shown for different groups. X axis: patient groups; Y axis: aortic calcification score.

The calcification score of patients with different age ranges (each range contains around 1/3 of total patients with each etiology) was shown in Fig. S1. For atherosclerotic patients, patients aged more than 65 years showed significantly higher calcification score compared to younger patients, whereas for infected etiology, patients aged more than 60 years would have significantly higher calcification score. For patients with immunological etiologies, patients aged 50 years or younger had significantly lower calcification score compared to older patients. Table 2 and Fig. 2 demonstrated the calcification characteristics of patients with different etiologies. For the total aorta, the atherosclerotic group had the highest level of calcification, while the immunological group had the lowest level (Fig. 2). The infrarenal aorta had the highest level of calcification for all types of lesions. For the ascending aorta, aortic arch, and descending aorta, the atherosclerotic group also had a significantly higher calcification score. The infected group had a higher level of calcification compared to healthy controls concerning visceral aorta, whereas the immunological group had a lower level of calcification compared to healthy controls for infrarenal aorta.

**Table 2:** Calcification and ulcer characteristics.

	Infected (n = 30)	Immunological (n = 34)	Atherosclerotic (n = 93)	Health (n = 160)
Position of ulcer/opening on the aorta				
Ascending aorta or arch, n (%)	1 (3.33)	9 (26.47)	27 (29.03)	NA
Thoracic, n (%)	3 (10.00)	8 (23.53)	22 (23.66)	NA
Visceral, n (%)	6 (20.00)	6 (17.65)	3 (3.23)	NA
Infrarenal, n (%)	20 (66.67)	8 (23.53)	30 (32.26)	NA
All aorta, n (%)	0	3 (8.82)	11 (11.83)	NA
Aortic calcification score	9.90 ± 1.21	6.12 ± 1.57	14.77 ± 0.90	8.11 ± 0.59
Ascending	0.67 ± 0.22	0.65 ± 0.26	1.40 ± 0.19	0.61 ± 0.10
Arch	1.30 ± 0.30	1.32 ± 0.40	2.72 ± 0.23	1.45 ± 0.13
Thoracic	1.70 ± 0.32	1.32 ± 0.39	3.06 ± 0.24	1.36 ± 0.15
Visceral	2.13 ± 0.29	1.03 ± 0.35	3.01 ± 0.25	1.39 ± 0.15
Infrarenal	4.10 ± 0.41	1.79 ± 0.43	4.58 ± 0.22	3.31 ± 0.20
Max calcification score of aortic parts	4.20 ± 0.40	2.24 ± 0.49	5.06 ± 0.19	3.57 ± 2.39
Calcification score of aortic ulcer part	3.67 ± 0.38	1.97 ± 0.46	4.29 ± 0.21	NA
Ulcer part with highest score, n (%)	18 (60.00)	12 (35.29)	56 (60.22)	NA

The aorta is divided into the ascending arch, descending, visceral, and infrarenal aorta. All aorta means ulcers are observed in more than one aortic parts.

**Figure 3:**
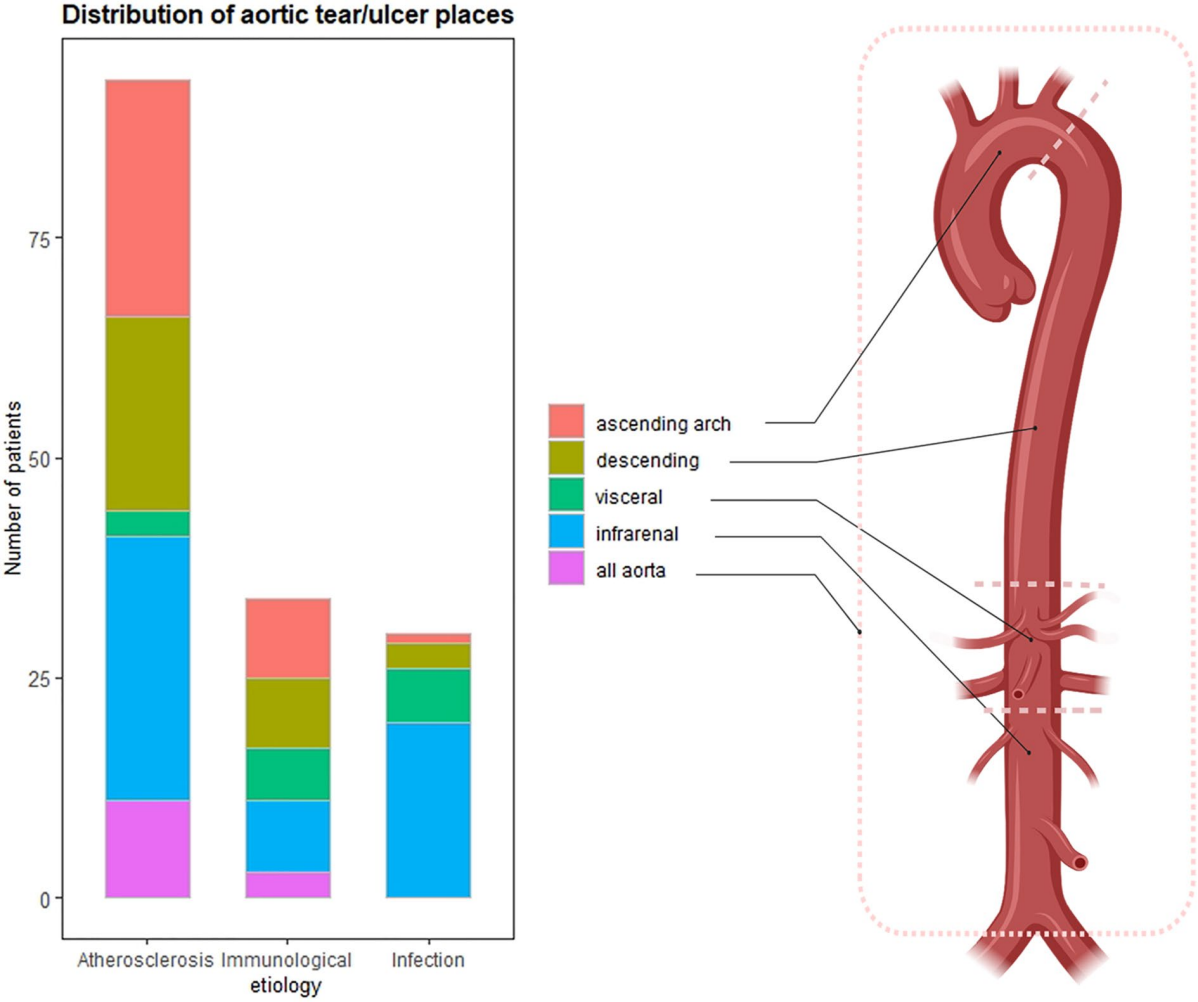
Distribution of aortic ulcers in the disease groups. X axis: etiology of PAU/pseudoaneurysm; Y axis: total number of patients. The aorta is divided into the ascending arch, descending aorta, visceral aorta, and infrarenal aorta. All aorta means ulcers are observed in more than one aortic parts.

Regarding the position of lesions, aortic tears/ulcers mostly occurred on the ascending aorta or arch (29.03%), descending aorta (23.66%), and infrarenal aorta for the atherosclerotic group (Fig. 3). The immunological group had a more even distribution, while most tears/ulcers took place at the infrarenal aorta (66.67%) for the infected group. Multiple tears/ulcers occurred for some immunological (8.82%) and atherosclerotic (11.83%) patients. As shown in Table 2, for patients of infected or atherosclerotic etiology, 60% (18/30) and 60.22% (56/93) of the tears/ulcers occurred at the aortic parts with the highest level of calcification.

### Risk factors for PAU/pseudoaneurysm or AAS of atherosclerotic etiology

Logistic regression was conducted to find the predictors of pseudoaneurysm or PAU occurrence in all subjects excluding immunologic or infected origins (Table 3). Hypertension, smoking, peripheral artery disease, as well as the longitudinal arch, longitudinal descending aortic, and cross-sectional infrarenal aortic calcification were risk factors for developing diseases, while diabetes was a protective factor. Patients with longitudinal calcification exceeding 1/3 of the aortic arch had an increased risk of approximately 13 times as high for acquiring diseases (OR 13.231, 95% CI 3.275–53.457). The presence of longitudinal calcification of the descending aorta or cross-sectional calcification of the infrarenal aorta had an approximate risk of 8.5 and 9 times higher for developing diseases (OR 8.484, 95% CI 3.020–23.831; OR 8.804, 95% CI 1.747–44.360). As illustrated in Table 3, logistic regression was also conducted to seek the predictors of AAS. After adjusting for age, longitudinal calcification of the descending aorta exceeding 1/3 length was found to be associated with AAS (OR 4.662, 95% CI 1.669–14.025).

**Table 3:** Logistic regression analysis for the risk factors of diseases in atherosclerotic patients (n = 253).

	Coefficient	OR	95% CI for OR	p
Risk factors of pseudoaneurysm or PAU				
Hypertension	1.261	3.529	1.535–8.113	0.003
Smoking	1.167	3.211	1.457–7.076	0.004
Peripheral artery disease	1.767	5.853	2.434–14.074	< 0.001
Diabetes mellitus	− 0.995	0.370	0.164–0.836	0.017
Calcification percentage				
Long-arch: 0–1/3	0.488	1.630	0.654–4.063	0.295
Long-arch: 1/3–2/3	2.583	13.231	3.275–53.457	< 0.001
Long-arch: 2/3–1	0.706	2.027	0.453–9.067	0.355
Long-descending: 0–1/3	2.138	8.484	3.020–23.831	< 0.001
Long-descending: 1/3–2/3	0.750	2.118	0.670–6.691	0.201
Long-descending: 2/3–1	1.613	5.018	1.054–23.892	0.043
Cross-infrarenal: 0–1/4	2.175	8.804	1.747–44.360	0.008
Cross-infrarenal: 1/4–1/2	− 0.281	0.755	0.224–2.548	0.651
Cross-infrarenal: 1/2–3/4	0.423	1.527	0.475–4.911	0.478
Cross-infrarenal: 3/4–1	− 1.160	0.313	0.115–0.853	0.023
Risk factors of AAS				
Age	− 0.065	0.937	0.896–0.981	0.005
Calcification percentage				
Long-descending: 0–1/3	1.026	2.790	0.974–7.995	0.056
Long-descending: 1/3–2/3	1.539	4.662	1.669–14.025	0.003
Long-descending: 2/3–1	1.584	4.875	1.701–13.969	0.003

Backward conditional method was used for logistic regression. The model had an AUC of 0.887 in ROC analysis.

## Discussion

Calcification occurs in both the intimal and medial layers of the aorta^16,17^. Regardless of its origin, calcification could alter the local mechanical and hemodynamic condition of the aorta, increasing the risk of atherosclerotic plaque, aneurysm rupture, dissection progression, as well as worsening prognosis after endovascular repair^18–21^. Currently, little has been reported on the role of calcification in pseudoaneurysms or PAU.

Aortic pseudoaneurysm is commonly secondary to trauma or iatrogenic injuries. Less frequently, aortic pseudoaneurysms could result from infections, autoimmune diseases, and PAU^14,22,23^. Regardless of its causes, pseudoaneurysm could be life-threatening and normally indicates prompt intervention. Similarly, PAU could present with severe clinical symptoms with a rupture rate of up to 40%^3^. However, asymptotic PAU with minimal growth was observed on incidental imaging findings^24^. In clinical practice, we noticed that despite distinct concomitant causes, the place of aortic tears/ulcers for aortic pseudoaneurysm and PAU was often located close to calcification. Thus, we sought to confirm and quantify this relationship using CT or CTA images. A set of patients receiving CT for health examination was also included for comparison.

Concerning calcification assessment systems, the Agatston score, first applied in the coronary artery, was widely accepted for calcium quantification and cardiovascular risk prediction^12,25^. Nevertheless, for the aorta, to better emphasize the place (e.g. the anterior or posterior) and characteristics (e.g. patchy or concentric) of calcification, various studies have proposed respective methods for different purposes^7,13,21,26^. Herein, we evaluated the status of aortic calcium based on the percentage of longitudinal and cross-sectional calcification of 5 segments of the aorta. Although it may not be as accurate as the Agatston score, the method was more convenient and applicable for both CTA and CT images. Since a percentage was calculated, the method could be adapted to images of different slice intervals. In addition, a higher number of infected or immunological aortic lesions are referred to our center each year^27^, making it possible to investigate and compare the role of calcification in pseudoaneurysm/PAU of different etiologies.

A higher rate of infected and immunological lesions received open or endovascular repair since most of these patients had pseudoaneurysms and presented symptoms. Patients with immunological etiology were younger with lesser cardiovascular comorbidities. An important confounding factor in calcification assessment is the age of subjects^5^. We observed that although patients of atherosclerotic origin had higher average calcification score, most of it was due to the age of these patients being > 65 years old. Patients with infected pseudoaneurysms had significantly increased levels of calcification at a younger age (60 years old). This result suggested that early aortic calcification may be a risk factor for infected aneurysm. Immunological lesions, on the other hand, had significantly elevated calcification score at > 50 years old. An increased level of inflammatory stimuli to the aortic wall may accelerate the calcifying speed in these patients and become a challenge to surgical planning^28^.

Apart from age, the general characteristics of total aortic calcification and its relationship with the location of aortic tears/ulcers were evaluated. Consistent with previous studies^13,29^, patients with different etiologies had the highest level of calcification at infrarenal aorta. Interestingly, infected lesions did not differ from atherosclerotic lesions at the abdominal aorta, which suggested that exacerbated abdominal calcification was a special characteristic of infected aneurysm. Another interesting finding was that immunological lesions only had a lower level of calcification at the infrarenal aorta compared to healthy controls. The level of calcification for the thoracic aorta may be higher for these patients than for healthy people in a similar age range. A similar distribution of aortic tear/ulcer location with calcification was observed. Around 2/3 of patients with infected or atherosclerotic etiologies had ulcers that occurred at the aortic parts with the highest level of calcification. Despite different distributions and underlying causes, calcification may correlate with pseudoaneurysm and PAU.

To further explore this correlation and exclude the impact of immunological/infected factors, we used logistic regression to evaluate the risk of calcification for PAU/pseudoaneurysm of atherosclerotic etiology. The result showed that longitudinal arch, longitudinal descending aortic, and cross-sectional infrarenal aortic calcification were risk factors for developing the lesions, and descending aorta longitudinal calcification was specifically associated with patients presenting with symptoms (AAS patients). Tanne et al. found that the presence of severe descending aorta calcification was the main predictor of ischemic cerebrovascular events (OR 4.9, 95% CI 1.8 to 13.5)^6^. Eisen et al. also noticed that thoracic aortic calcification was associated with an increased risk of death and cardiovascular disease for angina patients^30^. Calcification of the abdominal aorta was considered as an independent predictor of cardiovascular-related events or death in the general population^31^. Besides reflecting the increased burden of vascular (atherosclerotic) disease^32,33^, our study proposed that certain calcification patterns may weaken the aortic wall and be more prone to PAU or pseudoaneurysm formation. Patients with higher levels of these calcifications should be monitored to prevent acute aortic syndromes.

This study has some limitations. Calcification was measured manually without an automated system, which might introduce bias. Vessels apart from the aorta were not measured. In addition, since the images were only observed for hospitalized patients, whether calcification was the cause or result of pseudoaneurysm or PAU was still disputable. Additional subjects and longitudinal follow-up images are required to validate our findings and predict the impact of calcification on disease progression.

## Conclusion

In conclusion, tears/ulcers of pseudoaneurysm and PAU were both generally found at the part of the aorta with the highest level of calcification, and distinct aorta calcification characteristics were observed for lesions of different etiologies. Longitudinal arch/descending aortic and cross-sectional infrarenal aortic calcification were risk factors for acquiring the diseases, and AAS was associated with longitudinal calcification of the descending aorta.

### Supplementary Information

Supplementary Figure S1.

Supplementary Legends.

Supplementary Table S1.

## Data Availability

The datasets used and/or analysed during the current study available from the corresponding author on reasonable request.
