# Supplementary figures and images for: Aortic calcification correlates with pseudoaneurysm or penetrating aortic ulcer of different etiologies
Source: Sci Rep. 2024 Jan 2;14:25. doi: 10.1038/s41598-023-49429-y (PMC10761832; doi:10.1038/s41598-023-49429-y)

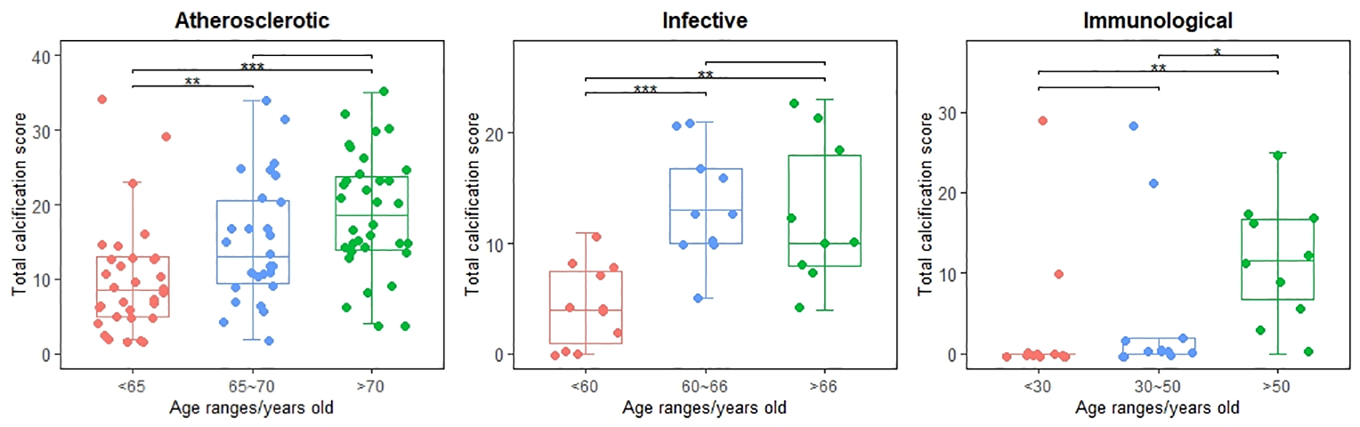

Supplement: Supplementary file 1 — Supplementary Figure S1. [file 41598_2023_49429_MOESM1_ESM.tif]
