# Supplementary material for: Aortic calcification correlates with pseudoaneurysm or penetrating aortic ulcer of different etiologies
Source: Sci Rep. 2024 Jan 2;14:25. doi: 10.1038/s41598-023-49429-y (PMC10761832; doi:10.1038/s41598-023-49429-y)
Supplement: Supplementary file 2 — Supplementary Legends. [file 41598_2023_49429_MOESM2_ESM.docx]

**Figure S1. Calcification score in different age ranges in patients with different etiologies.**

Each group of patients is divided into 3 age ranges, and the total aortic calcification score of patients is shown and compared. X axis: age ranges; Y axis: total aortic calcification score.
