# Supplementary material for: Aortic calcification correlates with pseudoaneurysm or penetrating aortic ulcer of different etiologies
Source: Sci Rep. 2024 Jan 2;14:25. doi: 10.1038/s41598-023-49429-y (PMC10761832; doi:10.1038/s41598-023-49429-y)
Supplement: Supplementary file 3 — Supplementary Table S1. [file 41598_2023_49429_MOESM3_ESM.docx]

**Table S1. Baseline characteristics for different diseases.**

|  | Pseudoaneurysm (n = 77) | PAU (n = 80) | Health (n = 160) | p |
| --- | --- | --- | --- | --- |
| Age, (mean years ± SD) | 55.51 ± 2.00 | 65.19 ± 1.28 | 67.64 ± 0.63 | <.001 |
| Sex, male n (%) | 61 (79.22%) | 66 (82.50%) | 129 (80.63%) | .897 |
| Symptom, n (%) | 60 (77.92%) | 24 (30.00%) | NA |  |
| Disease course, (mean months ± SD) | 4.82 ± 1.75 | 8.70 ± 2.07 | NA | .153 |
| Hypertension, n (%) | 41 (53.25%) | 61 (76.25%) | 77 (48.13%) | <.001 |
| Coronary heart disease, n (%) | 12 (15.58%) | 40 (50.0%) | 28 (17.50%) | <.001 |
| Stroke, n (%) | 10 (12.99%) | 19 (23.75%) | 21 (13.13%) | .083 |
| Diabetes Mellitus, n (%) | 13 (16.88%) | 19 (23.75%) | 56 (35.00%) | .009 |
| Dyslipidemia, n (%) | 11 (14.29%) | 29 (36.25%) | 66 (41.25%) | <.001 |
| Ever smoke, n (%) | 37 (48.05%) | 53 (66.25%) | 63 (39.38%) | <.001 |
| Ever drink, n (%) | 19 (24.68%) | 31 (38.75%) | 59 (36.88%) | .120 |
| Peripheral artery disease, n (%) | 42 (54.55%) | 69 (86.25%) | 85 (53.13%) | <.001 |
| Etiology, n (%) |  |  |  | <.001 |
| Infection, n (%) | 30 (38.96%) | 0 | NA |  |
| Immunological, n (%) | 22 (28.57%) | 12 (15.00%) | NA |  |
| Atherosclerotic, n (%) | 25 (32.47%) | 68 (85.00%) | NA |  |
| Position of ulcer/opening on the aorta |  |  |  | .002 |
| Ascending aorta and arch | 18 (23.38%) | 19 (23.75%) | NA |  |
| Thoracic | 13 (16.88%) | 20 (25.00%) | NA |  |
| Visceral | 14 (18.18%) | 1 (1.25%) | NA |  |
| Infrarenal | 29 (37.66%) | 29 (36.25%) | NA |  |
| All aorta | 3 (3.90%) | 11 (13.75%) | NA |  |
| Aortic calcification score | 9.40 ± 1.01 | 14.44 ± 0.97 | 8.11 ± 0.59 | <.001 |
| Ascending | 0.79 ± 0.18 | 1.39 ± 0.20 | 0.61 ± 0.10 | .001 |
| Arch | 1.70 ± 0.25 | 2.58 ± 0.25 | 1.45 ± 0.13 | <.001 |
| Thoracic | 1.87 ± 0.25 | 2.96 ± 0.27 | 1.36 ± 0.15 | <.001 |
| Visceral | 1.90 ± 0.24 | 2.91 ± 0.26 | 1.39 ± 0.15 | <.001 |
| Infrarenal | 3.14 ± 0.29 | 4.60 ± 0.25 | 3.31 ± 0.20 | <.001 |
| Operation |  |  |  | <.001 |
| Open, n (%) | 6 (7.79%) | 3 (3.75%) | NA |  |
| Endovascular, n (%) | 52 (67.53%) | 27 (33.75%) | NA |  |
| Observation, n (%) | 19 (24.68%) | 50 (62.50%) | NA |  |

NA: not avaliable. SD: standard deviation.
